# Connecting the dots: A practical evaluation of web-tools for describing protein dynamics as networks

**DOI:** 10.3389/fbinf.2022.1045368

**Published:** 2022-10-19

**Authors:** Francesco Petrizzelli, Tommaso Biagini, Salvatore Daniele Bianco, Niccolò Liorni, Alessandro Napoli, Stefano Castellana, Tommaso Mazza

**Affiliations:** ^1^ Bioinformatics Laboratory, Fondazione IRCCS Casa Sollievo della Sofferenza, San Giovanni Rotondo, Italy; ^2^ Department of Experimental Medicine, Sapienza University of Rome, Rome, Italy

**Keywords:** protein structure networks, molecular dynamics simulations, network analysis, graph theory, dynamic residue interaction networks

## Abstract

Protein Structure Networks (PSNs) are a well-known mathematical model for estimation and analysis of the three-dimensional protein structure. Investigating the topological architecture of PSNs may help identify the crucial amino acid residues for protein stability and protein-protein interactions, as well as deduce any possible mutational effects. But because proteins go through conformational changes to give rise to essential biological functions, this has to be done dynamically over time. The most effective method to describe protein dynamics is molecular dynamics simulation, with the most popular software programs for manipulating simulations to infer interaction networks being RING, MD-TASK, and NAPS. Here, we compare the computational approaches used by these three tools—all of which are accessible as web servers—to understand the pathogenicity of missense mutations and talk about their potential applications as well as their advantages and disadvantages.

## Introduction

Molecular dynamics (MD) simulation is one of the most effective methods for assessing how a system changes over time and is regarded as the most effective computing tool for this purpose, particularly in physics and chemistry (Karplus and McCammon 2002). It has also had great success in biology over the past 10 years, where it has frequently been employed to test hypotheses and, as a result, aid in providing specific answers to questions about the structural characteristics and dynamical mechanisms of biological systems (Biagini et al., 2017), like the impact of disease mutations on the protein functionality. Nowadays, we are dealing with a dramatically increased quantity and quality of simulation data due to improvements in hardware (Biagini et al., 2019) and software, as well as more frequent use of enhanced sampling techniques. This comes along with the need for new and powerful analysis tools, capable of not only extracting information but also capturing key-properties fundamental to large-scale conformational changes (Melo et al., 2020).

One of these tools is the Protein Structure Network (PSN) (Greene 2012), which can model the spatial organization of proteins and record long-distance structural communications. In this model, nodes stand in for amino acids and are connected by edges, which can either represent the physical interactions between two residues or their spatial separation (Residue Interaction Network, RIN, or Protein Contact Network, PCN). The benefit of employing such a “nodes-edges” representation is that it makes it possible to resort to Graph Theory to analyze MD simulation results (del Sol et al., 2006), with the exploitation of a broad range of local (Borgatti 2005; Borgatti and Everett 2006; Mazza et al., 2012) (i.e., regarding nodes or edges) and global (Lozares et al., 2015; Mazza et al., 2010; Menniti et al., 2013) (i.e., regarding the entire network) network metrics to identify topologically important hubs, e.g., nodes fundamental for graph connectivity (betweenness) or identify residues located in functional regions (closeness). This aspect is brilliantly presented in (Liang et al., 2020), where the authors provided a thorough overview of protein network approaches and a description of the tools that are currently available for converting protein structures into graphs, with the aim of providing a new level of insight into seemingly unpredictable systems (Barabasi and Albert 1999). It has a history of successful applications: from the graph spectral methods used by (Kannan and Vishveshwara 1999) to identify side-chain clusters to the characterization of more complex molecular mechanisms (Karamzadeh et al., 2017), with important acknowledgements in drug design (Brown and Bishop 2017) and in the evaluation and prediction of disease mutations (Cheng et al., 2008; Doshi et al., 2016).

## How to turn a molecular dynamics trajectory into a network

The ability to build a network from conformational ensembles, such as snapshots taken from MD simulations, is crucial for accounting for those links that form or break as time progresses. Starting from a trajectory file, which is a collection of 3D coordinates of a protein structure in each of the various conformations investigated during the simulation time, we can either analyze the network properties of individual protein structures corresponding to each trajectory frame or work on the average structure derived over the course of the MD simulation to account for these atomic fluctuations.

Recent years have witnessed the development of several tools that integrate MD simulation data. The majority of these tools build simple unweighted PCNs using a geometry-based methodology, which consists of defining the contacts between alpha/beta carbon pairs (C_α_, C_β_) or between centroids of the amino acids of a protein. Contacts are established if such elements are within a predefined cut-off distance. This distance threshold, which typically ranges between 4.5 and 8.5 Å (Viloria et al., 2017), was carefully selected to map connections only for non-covalent intramolecular interactions, avoiding networks that are either poorly or excessively connected. When these networks are obtained from MD data, they are referred to as Dynamical Network Models (DNMs) or Dynamic Residue Interaction Networks (DRINs), and two nodes are connected only if their distance is less than a cut-off value in the range reported above for at least 65% of the simulation time. The advantage of creating these dynamic networks is that their properties, like dynamic residue-residue cross-correlations or their interaction frequencies, could be assigned as weights to the edges, providing a more accurate description of the system (Sethi et al., 2009).

Here, we evaluate and contrast a few tools made to summarize MD trajectories in a network, providing our general opinion on the benefits and drawbacks of each strategy. Many of these tools are standalone software packages (Felline et al., 2022), usually easy to use but requiring some technical knowledge to install and configure. In order to test their adaptability and usability (Table 1) for the specific task of assisting in the interpretation of the role of missense mutations in conformational changes, we only kept those that are available as web servers, i.e., NAPS, MD-TASK, and RING. This was done because one of the goals of this mini-review was to support widespread usage of this class of tools.

**TABLE 1:** Web-tools for analyzing MD trajectories as networks. For each tool, we summarize the required input formats, different topologies that can be used to build a network, and which network centrality indices are computed. Finally, the main strengths of each tool are highlighted.

Tools	Url	Standalone	Trajectory requirements	Main strength
	*Network topology*		*Output - network centralities*	
NAPS	https://bioinf.iiit.ac.in/NAPS/	No	DCD format max 50 frames	Multiple network types
	*Cα, Cβ, geometric center and center of mass distances* ^*^ *, energy*		*Weighted/unweighted degree, shortest path, closeness, betweenness, clustering coefficient, eigenvector centrality, eccentricity, average nearest neighbor degree and edge betweenness*	
**MD_TASK**	https://mdmtaskweb.rubi.ru.ac.za/	Yes	Multiple MD formats max 250 Mb	Great MD format support
	*Cβ (Cα for Glycine) distances* *		*Betweenness, degree, eccentricity, averaged shortest paths, closeness, Katz, PageRank and eigencentrality*	
**RING3.0**	https://ring.biocomputingup.it/	Yes	PDB and mmCIF format max 200 Mb	Probabilistic residue interaction networks
	*Closest atoms, lollipop (Center of mass), Cα or Cβ of residues connected by multiple interaction type*		*Cytoscape-compatible (.json) output format*	

* distances ≤ *user defined threshold* (*authors usually suggest 6.5–7.5 Å to avoid disconnected/highly connected graph*)

## Network based analysis of protein structures

Network based Analysis of Protein Structures (NAPS) is an online tool available at https://bioinf.iiit.ac.in/NAPS/. It was originally built for the analysis and interactive visualization of PCN or RIN derived from static single proteins or protein complexes (Chakrabarty and Parekh 2016). Its key characteristic is the creation of various network types, both unweighted and weighted, from a single PDB, using C_α_, C_β_, geometric center, or center of mass distances to draw edges between residues. These features were greatly expanded in NAPS 2.0 (Chakrabarty et al., 2019) with the possibility to analyze MD trajectories, exported as *. dcd* files, and represent them as average networks of the ensemble of trajectories, dynamic cross-correlations (DCC), and bipartite networks. The web tool offers the analysis of various centrality measures (with distance-based weights or unweighted) computed using igraph (Csardi and Nepusz 2006) and NetworkX (Hagberg et al., 2008): *degree, average shortest path, closeness and betweenness, clustering coefficient, eigenvector centrality, eccentricity, average nearest neighbor degree* (ANN degree), and *edge betweenness,* in addition to standard global properties, i.e. *number of nodes and edges, diameter, clustering coefficient, average degree,* and *average path length*. These can be computed for the network representing a specific simulation frame or for an “average network”, i.e., an individual network wiring nodes with edges only if the interactions they represent last more than 60% (default value) of the entire simulation time. This latter option is available by choosing “Ensemble Analysis” on the submission form, whereas “Timestep Analysis” allows users to compute and compare the centrality values pertaining to a particular time step to either those of other time steps or to those of the average network. Similarly, the shortest path can be obtained and compared for any pair of residues.

While we considered NAPS to be one of the most comprehensive web tools currently available in terms of options for network construction and analysis, we also ran into a number of usability issues, mostly pertaining to the MD section. In particular, the user is forced to limit the trajectory to 50 frames even though the web tool is intended to handle large MD trajectory files. Additionally, there are a few minor bugs in the web server that restrict user experience. We observe, in fact, numerous glitches when structures with more than 1000 residues are submitted, along with errors when ligands or heteroatoms are present in the input topology. The introduction of a standalone version and a more potent web-server would both be highly advantageous.

## MD-TASK

MD-TASK is a Python-based suite that can generate residue interaction networks from MD simulations and uses NetworkX functions to compute average residue network centrality metrics. It is available as a downloadable program as well as a web server (https://mdmtaskweb.rubi.ru.ac.za/), where it is integrated with the MODE-TASK suite, a normal mode and essential dynamics analysis toolkit (Brown et al., 2017; Amamuddy et al., 2021). Remarkably, MD-TASK supports many different MD file formats, including the most commonly used by AMBER (*.netcdf*), NAMD (*.dcd*) and Gromacs (*.xtc*) simulation frameworks. DRINs can be constructed using these trajectory files, with single residues as nodes and edges drawn when the distance between C_β_ atoms (C_α_ for Glycine) of two residues meets a user-defined cut-off (usually 6.5–7.5 Å).

It is noteworthy that the metrics are not computed on average networks, but rather the software returns changes in eight different centrality metrics to residues over a trajectory, which are obtained by aggregating the mean, median, and standard deviation of each frame’s residue metrics. The values that are obtained can either be displayed in the 3D structure with a color gradient or included in a downloadable *csv* file. MD-TASK also enables the construction of a weighted residue contact map from a trajectory, which is a weighted network graph with edges between a residue of interest and the other residues that are weighted in accordance with how frequently the interactions occur. The output is another network centered on the residue of interest and surrounded by the residues it interacts with. Finally, besides DRIN, the MD-TASK tool suite also deals with DCC and perturbation response scanning (PRS) techniques (Atilgan and Atilgan 2009).

When it comes to usability, we found MD-TASK to be fairly simple, with an easy-to-use submission form, a straightforward but sharp output visualization, and the option of direct comparison with previously submitted jobs. The developers have provided a simple tool (https://github.com/oliserand/MD-TASK-prep) that makes it possible to reduce the trajectory size by keeping only heavy atoms, greatly increasing the number of frames that can be submitted. The 250 Mb limit for the trajectory size is undoubtedly a limitation, but it is partially overcome by this tool. The MD-TASK results page, on the other hand, is accompanied by a color legend, which helps identify significant regions or domains. However, the data interpretation remains difficult without prior knowledge of the altered molecular mechanisms.

## Residue interaction network generator

Residue Interaction Network Generator (RING) offers a simple way to build a network starting from an MD trajectory. The edges connecting the nodes are atom-specific physico-chemical interactions, such as disulfides, salt bridges, hydrogen bonds, aromatic interactions, or more general van-der-Waals contacts between residues. These interactions also rely on the concept of “distance”, computed using only geometrical criteria and after an exhaustive analysis of the entire PDB content (116568 X-ray and NMR structures as of April 2016). To represent strict and permissive parameters, two different distance thresholds were selected; the pair [2.84, 2.87] Å, for example, corresponds to interactions that stabilize the packing of different secondary structure elements, i.e., bridges between alpha-helices or turns; similar values for the main chain hydrogen bonds at [2.94, 2.98] correspond to interactions between adjacent strands in β-sheets, whereas [5.01, 5.04] Å identifies bonds in α-helices separated by a turn. Beyond 5.6 Å, only spurious interactions are identified (Piovesan et al., 2016).

The most recent RING 3.0 version (Damiano et al., 2022) is available as a standalone package or in a completely redesigned web-portal at https://ring.biocomputingup.it/. It can process molecular dynamics simulations as multi-state files (in PDB and mmCIF format, up to 200 MB in size). Users can choose between four alternative types of network, and interactions involving the same residue but different atoms are sorted by energy and distance. In this case, the user may choose to retrieve only the most energetic interaction, a multigraph with all the interactions, or only one interaction for each type. For each node, a number of structural attributes are reported, including secondary structure, vertex degree (the number of directly connected nodes), experimental uncertainty for X-ray structures, conformational energy preferences, conservation (Shannon entropy), and cumulative mutual information (MI). From these, RING now creates probabilistic networks that take into account the frequency of connectivity between states (or snapshots): the edges have an associated weight (range: [0–1]) that represents the frequency at which the interaction was present in the conformational ensemble. Finally, RING returns an interactive graph and a network that can be downloaded in a format, that is, optimized for Cytoscape. This dynamic layout makes it possible to quickly and effectively identify functional residues.

We consider RING to be by far the most interesting approach, encompassing different levels of granularity in the network construction. The output page, on the other hand, focuses primarily on the identification and description of significant interactions and does not include any analysis strategy based on network topological metrics that could aid the user in the localization of hotspots. Finally, the PDB format, which was probably chosen to speed up computation, poses a significant limitation due to its large size, thereby forcing the user to rely on the standalone version even for small trajectories.

## How to use molecular dynamics-networks to understand the role of missense mutations?

In the last decade, molecular dynamics has become one of the most widely used computational approaches for the generation of hypotheses regarding the impact of missense mutations. From a network point of view, when these perturbations affect the most central nodes, we may observe a disruption in the transmission of information, with consequences for protein stability and the alteration of fundamental protein functions. There are several methods that can be used to investigate this aspect, such as PRS (Atilgan and Atilgan 2009) or comparing centrality metrics gleaned from various trajectory comparisons.

All of the tools mentioned above have been successfully used to achieve this goal. However, a performance-based evaluation poses conceptual difficulties because each approach might take into account different altered mechanisms (such as local impact and long-range effects) and, as a result, a direct comparison is unreliable or even impossible. Moreover, given the fact that fluctuation correlation is very hard to converge in molecular dynamics simulations (Hospital et al., 2015), it is often the case that the dynamical network is trajectory dependent or simulation time dependent, leading to inconclusive statements. (Li et al., 2019).

Here, to overcome these issues, we focused on the description of the ability of each strategy to identify key nodes and capture the impact caused by pathogenic mutations using already extensively described MD trajectories. We specifically used the wild-type and mutant trajectories of the catalytic Jumonji (JmjC) domain of KDM6A, a known cancer driver gene and the gene responsible for type 2 Kabuki Syndrome, to show how a particular set of missense mutations can be connected to the impairment of the interaction between the protein and the interacting protein (Petrizzelli et al., 2020; Biagini et al., 2022).

In Supplementary Table S1 *,* we report all the centrality values computed by each tool for the wild-type (WT) and one of the most detrimental mutations, Arg1255Trp (R1255W). Default options were selected: NAPS “*Ensemble Analysis*” on C_α_ distances was executed on 50 frames extracted from each trajectory; on the contrary, MD-TASK mean centralities were computed on C_β_ distances of 1000 frames obtained by taking advantage of the “trajectory-cutting” MD-TASK-prep tool. Finally, since RING does not offer metric analysis, we extracted the dynamic interaction network computed on 300 frames and used RINalyzer (Doncheva et al., 2011) of Cytoscape to compute the shortest path betweenness, closeness, and degree centralities using frequency as edge weight. Then, we examined the top 15% nodes for each metric, using a metric-specific color scale, with a special emphasis on the betweenness centrality (BC), the most widely used measure of centrality. In fact, betweenness measures how many shortest paths between any two nodes of a network pass through a given node given the total number of shortest paths in the network and, then, furnishes a measure of the overall importance of the given node.

**FIGURE 1:**
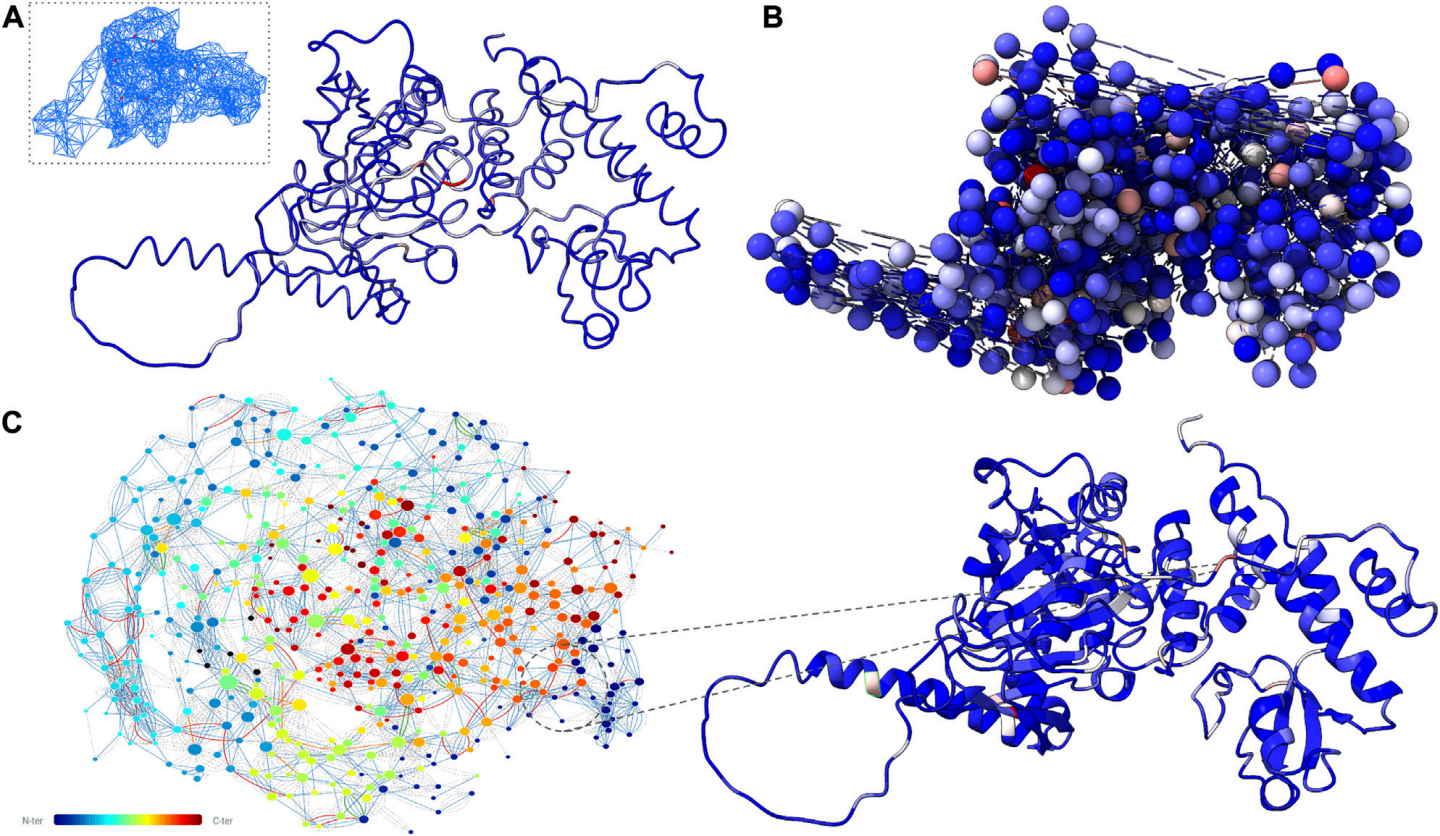
Networks produced on wild-type KDM6A trajectory data using three different methods. Tool-specific betweenness values were mapped onto the corresponding protein representation using a color scale ranging from blue (low) to red (high). **(A)** NAPS Ensemble network with C_α_ distances as edges: C_α_-representation of the KDM6A protein with, in the box, an example of a network downloadable from the web-server. **(B)** “Spacefill” representation of the KDM6A protein with contacts computed by MD-TASK using C_β_ distances. **(C)** Left: dynamic residue interaction network obtained using RING, with residue-number color scheme. Right: Cartoon representation of all-atom KDM6A protein.

Comparing the BC values of the missense variants located within the Jumonji domain (Supplementary Table S1 - DiseaseMutation), we found that NAPS and MD-TASK identify as central some of these well-known pathogenic hotspots, which is consistent with the enrichment of central residues in the protein’s core shown in Figure 1A,B, while none of these amino acidic positions but 1049 can be considered central in the interaction network obtained using RING. However, the poor correlation observed between pathogenic sites and central nodes could be mainly ascribed to the fact that their central role in structural rearrangements is not enough to determine the impact of a mutation on that site and supports the need for further attributes that account for the functional role of each residue, like evolutionary information (Liang et al., 2020).

On the other hand, we found that many wild-type top residues for all the generated networks had decreased betweenness when comparing the 100 top nodes between wild-type and R1255W trajectories. With a significant reduction in this region’s betweenness across all the R1255W metrics (Supplementary Table S1 *- RING*), RING, in particular, was able to highlight the linker domain’s central function more than the other tools (Figure 1C). This is consistent with our earlier observations that the alteration of the wild-type conformational transitions was correlated with the loss of fundamental hydrogen bonds between the linker domain and its surrounding region.

In conclusion, each tool retains its unique characteristics, with networks produced by NAPS and MD-TASK able to prioritize those residues essential for the functionality of the protein as discernible from the enrichment of mutant hotspots in their central residues, and networks produced by RING able to identify the specific altered mechanism between wild-type and mutant dynamics.

## Final thoughts and future plans

“*Different packages can have different niche strengths, and their strengths are often complementary.*” - M. Hucka.

Creating network-based methods to model molecular systems is a rich area of study that capitalizes on several research fields, mainly transcriptomics, proteomics, and ecology, as well as in computer science applied to biological sciences (Mazza et al., 2016; Piepoli et al., 2012; Mazzoccoli et al., 2016; Palmieri et al., 2020; Mazza et al., 2017; Capocefalo et al., 2018; Ballarini et al., 2009; Franco et al., 2006). However, these methods become especially fascinating when they are made to handle the massive amounts of data produced by enhanced or long-timescale MD simulations where, thanks to their vast potential, could help answer a variety of different questions, e.g., putative allosteric mechanism or protein-ligand interaction pathway, or also could be useful for challenging tasks like the identification of epistatic mutant sites (Castellana et al., 2015; Castellana et al., 2017;^2021^).

An ideal “MD to network” tool should be able to work with a variety of input formats, build trustworthy networks, analyze those networks’ topologies, and finally, provide simple visualization and interpretation of the results (Guzzi et al., 2022). Here, we provide a practical evaluation of the usability and applicability of three of the most popular web tools, NAPS, MD-TASK, and RING, to construct and analyze MD-based graphs. First of all, we would like to acknowledge the tremendous effort that has gone into developing and maintaining these web servers. We found them easy to use, even for beginners. But all of them have minor flaws, primarily because of the restrictions on input formats and size. When available, their standalone versions perform significantly better, but they cannot be proficiently used without the necessary programming skills and computing power. In addition, each software package exhibits a unique characteristic in the network construction, with varying degrees of granularity and specialization, and it offers the computation of the most common centrality metrics. For the identification of functionally significant residues, they all heavily rely on network/protein visualization, with many centrality metrics that can be either highlighted on the structure or downloaded as a file in both MD-TASK and NAPS.

Finally, focusing on applicability, we discovered that each software was able to identify “central” residues in functional domains with varying sensitivity and that the DRINs obtained by RING were the ones that best captured the disruption of fundamental interactions during the mutant trajectory and the impact on the dynamics of harmful mutations. However, it can be challenging to determine which metric, or set of metrics, might be the most sensitive in describing the investigated functional mechanism, and we discovered a general lack of statistical consistency, with little that has been proposed to serve as a guide for selecting metrics or as evidence for comparing them (Foutch et al., 2021). For instance, application of comparative evaluation with synthetic networks may aid in assessing the strength of the community structure by determining the informational value of a metric or when the combination of multiple metrics would be advantageous (Oldham et al., 2019; Rajeh et al., 2021).

In conclusion, we believe that integrating various approaches is essential to more effectively exploring the information contained in the topology of an MD-based network. Additionally, the integration of conservation and evolutionary attributes could significantly enhance this information, and the use of more sophisticated metrics, such as key-player and group-centrality metrics, could support the agnostic identification of central “hubs,” or structured/regulatory regions (Dassi et al., 2012) necessary for the functionality of the protein, that could previously only be identified thanks to prior knowledge obtained from experimental or literature studies (Parca et al., 2020).
